# Nur77 and PPARγ regulate transcription and polarization in distinct subsets of M2-like reparative macrophages during regenerative inflammation

**DOI:** 10.3389/fimmu.2023.1139204

**Published:** 2023-03-03

**Authors:** Éva Garabuczi, Nastaran Tarban, Éva Fige, Andreas Patsalos, László Halász, Tímea Szendi-Szatmári, Zsolt Sarang, Róbert Király, Zsuzsa Szondy

**Affiliations:** ^1^ Department of Integrative Health Sciences, Institute of Health Sciences, Faculty of Health Sciences, University of Debrecen, Debrecen, Hungary; ^2^ Doctoral School of Molecular Cell and Immune Biology, Faculty of Medicine, University of Debrecen, Debrecen, Hungary; ^3^ Doctoral School of Dental Sciences, Faculty of Dentistry, University of Debrecen, Debrecen, Hungary; ^4^ Department of Medicine, Johns Hopkins University School of Medicine, Institute for Fundamental Biomedical Research, Johns Hopkins All Children’s Hospital, St. Petersburg, FL, United States; ^5^ Department of Biological Chemistry, Johns Hopkins University School of Medicine, Institute for Fundamental Biomedical Research, Johns Hopkins All Children’s Hospital, St. Petersburg, FL, United States; ^6^ Department of Biophysics and Cell Biology, Faculty of Medicine, University of Debrecen, Debrecen, Hungary; ^7^ Department of Biochemistry and Molecular Biology, Faculty of Medicine, University of Debrecen, Debrecen, Hungary; ^8^ Section of Dental Biochemistry, Department of Basic Medical Sciences, Faculty of Dentistry, University of Debrecen, Debrecen, Hungary

**Keywords:** macrophage, PPAR gamma, Nur77, polarization, cardiotoxin, skeletal muscle injury, efferocytosis

## Abstract

Macrophage polarization is a process whereby macrophages develop a specific phenotype and functional response to different pathophysiological stimuli and tissue environments. In general, two main macrophage phenotypes have been identified: inflammatory (M1) and alternatively activated (M2) macrophages characterized specifically by IL-1β and IL-10 production, respectively. In the cardiotoxin-induced skeletal muscle injury model bone marrow-derived macrophages (BMDMs) play the central role in regulating tissue repair. Bone marrow-derived monocytes arriving at the site of injury differentiate first to M1 BMDMs that clear cell debris and trigger proliferation and differentiation of the muscle stem cells, while during the process of efferocytosis they change their phenotype to M2 to drive resolution of inflammation and tissue repair. The M2 population is formed from at least three distinct subsets: antigen presenting, resolution-related and growth factor producing macrophages, the latest ones expressing the transcription factor PPARγ. Nuclear receptor subfamily 4 group A member 1 (NR4A1; also termed Nur77) transcription factor is expressed as an early response gene, and has been shown to suppress the expression of pro-inflammatory genes during efferocytosis. Here we demonstrate that (1) Nur77 null BMDMs are characterized by elevated expression of PPARγ resulting in enhanced efferocytosis capacity; (2) Nur77 and PPARγ regulate transcription in different subsets of M2 skeletal muscle macrophages during muscle repair; (3) the loss of Nur77 prolongs M1 polarization characterized by increased and prolonged production of IL-1β by the resolution-related macrophages normally expressing Nur77; whereas, in contrast, (4) it promotes M2 polarization detected *via* the increased number of IL-10 producing CD206^+^ macrophages generated from the PPARγ-expressing subset.

## Introduction

Clearance of apoptotic cells (efferocytosis) by macrophages plays a central role in maintaining tissue homeostasis. During the efferocytosis process macrophages not only clear apoptotic cells and degrade them, but also release various biologically active signaling molecules the release of which is triggered by the apoptotic cell uptake itself. If these molecules are released in resting tissues, they provide local trophic support, as some of them are growth factors (1). In the thymus they contribute to the thymic selection processes by directing the formation of regulatory T cells (2), and by regulating the signalling threshold of negative selection (3). Following tissue injury, however, they drive the resolution of inflammation and tissue repair (4). Proper efferocytosis regulates the proper production of these engulfing macrophage-derived regulatory molecules. The efferocytosis process *in vivo* is initiated by finding the apoptotic cells by macrophages *via* the help of ‘find me’ signals released from the apoptotic cells (5). Macrophages then recognize the apoptotic cells *via* their characteristic cell surface changes. The most well-known cell surface change is the exposure of phosphatidylserine (PS), a key ‘eat me’ signal, on the surface of dying cells (6). Multiple receptors on the surface of macrophages such as Tim4, stabilin-2, or BAI1, recognize and bind to PS directly, whereas other receptors use bridging molecules to link the phagocytic receptor to PS (7). Bridging molecules are present in the serum but are also actively produced by the macrophages. One of them is milk fat globule-EGF-factor 8 (MFG-E8) which uses its RGD motif within its EGF-like domain to bind to various integrin receptors, such as β3 and β5 participating in the phagocytosis process, but also contains gamma carboxylated glutamate side chains to link the protein to PS (8, 9). In addition to using bridging molecules, integrin receptors also require coreceptors, such as Tim4 by integrin β1 (10) or transglutaminase 2 (TG2) and CD36 (11–13) by integrin β3, for their proper phagocytic function. Phagocytes in different tissues express different combinations of the various efferocytosis receptors, but all of the expressed ones assemble and function together in the phagocytic synapse to mediate tethering, and to initiate sufficient engulfment signaling once macrophages interact with the apoptotic cells (14). So far two efferocytosis signaling pathways have been identified that trigger the apoptotic cell uptake, and both lead to the activation of the small GTPase Rac1 (15). Nur77 (NR4a) is a transcription factor, which belongs to the steroid/thyroid hormone receptor superfamily, and is an orphan receptor for which no ligand is known (16). Besides forming a heterodimer with the retinoid X receptor to mediate retinoic acid-dependent transcription to reporters containing the DR5 regulatory element, it can also bind in monomeric form to promoters containing the Nur77 binding response element (17), as well as a homodimer to the Nur77 response element carrying ones (18). In addition, similar to other members of this transcription family, it is able to interact with other transcription factors, such as nuclear factor kappa-light-chain-enhancer of activated B cells (NF-κB) (19), and regulate their transcriptional activity. The activity of Nur77 was shown to be controlled through transcriptional regulation, posttranslational modifications, protein-protein interactions and subcellular localization (20, 21). Nur77 is abundantly expressed in various tissues including myeloid cells. Loss of Nur77 in macrophages has been reported to result in enhanced pro-inflammatory cytokine release following Toll like receptor 4 stimulation, and in an M1 type pro-inflammatory polarization in various atherosclerosis models. The effect has been linked to uncontrolled NF**-**κB activation leading to enhanced transcription of various inflammation-related genes (22). Recently the transcriptome of bone marrow-derived macrophages (BMDMs) from wild-type (WT) and Nur77-knockout (Nur77 KO) mice has been analyzed (23). In addition to the enhanced expression of a group of inflammation-related genes, IPA Upstream Regulator Analysis revealed Rac1 as an activated upstream regulator possibly mediating changes in gene expression induced by the loss of Nur77. Since enhanced Rac1 activity of BMDMs is known to be associated with enhanced efferocytosis capability, we decided to investigate phagocytosis of apoptotic cells mediated by the Nur77 KO macrophages. As phagocytosis of apoptotic cells plays a crucial role also in promoting formation of M2-like reparative macrophages which guide tissue repair during regenerative inflammation (24), we also followed the formation of reparative macrophages in the absence of Nur77 in the cardiotoxin–induced injury model of skeletal muscle.

## Results

### Nur77 KO bone marrow derived macrophages have enhanced efferocytosis capacity due to an increased integrin β3 and β5 signaling

**Figure 1:**
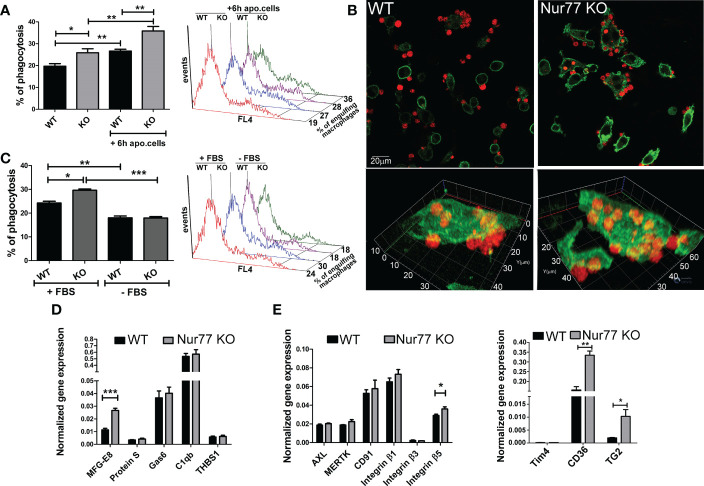
Loss of Nur77 in BMDMs leads to enhanced efferocytosis due to an increased integrin β3 and β5 signaling. **(A)** *In vitro* uptake of fluorescently-labeled apoptotic thymocytes by WT and Nur77 KO BMDMs following 24 h incubation after seeding the BMDMs or after 6h co-culture with non-labeled apoptotic thymocytes followed by additional 18 h incubation. The percentage of engulfing macrophages was determined by flow cytometry analysis. A representative histogram of each experimental setting is also shown. **(B)** One representative confocal image showing efferocytosis by wild-type and Nur77 KO BMDMs following 24h in culture. Alexa Fluor 488 conjugated anti-F4/80 antibody -labelled macrophages appear as green cells, while Deep Red dye-labelled apoptotic thymocytes appear as red cells. **(C)** Efferocytosis by WT or Nur77 KO BMDMs following 24h culture in 10% FBS containing DMEM or washing the culture medium away and using a fresh DMEM without FBS during the 45 min efferocytosis. A representative histogram of each experimental setting is also shown. **(D, E)** mRNA expressions of various bridging molecules and phagocytic receptors determined by qRT-PCR in WT and Nur77 KO BMDMs following 24 h in culture. β-actin was used as a reference gene. All the results are expressed as mean ± SEM (n = 3). Asterisks indicate statistical significance (*P < 0.05, **P < 0.01, *** p < 0.001).

Since previous studies indicated that Nur77 KO BMDMs have an enhanced Rac1 gene signature (23), and Rac1 is known to play a determining role also in efferocytosis by regulating lamellopodia formation (25), we checked whether Nur77 KO BMDMs have an altered efferocytosis capacity. As seen in **Figure 1A**, in line with the enhanced Rac1 gene signature, we also observed an increased efferocytosis capacity of BMDMs after a 45 min exposure to apoptotic cells. Loss of Nur77 resulted not only in an increase in the percentage of engulfing macrophages but also an increase in the number of engulfed apoptotic cells (**Figure 1B**) detected as an increased mean fluorescence within the engulfing macrophage population (415 ± 18 versus 482 ± 18 in the wild-type and knock out macrophages, respectively. Significantly different p<0.05). This enhancement was more pronounced, if macrophages engulfed apoptotic cells first for 6 h, and their efferocytosis capacity was determined 18 h later (**Figure 1A**). To check whether the observed difference in the efferocytosis capacity is related to a bridging molecule-driven efferocytosis pathway in Nur77 KO macrophages, their phagocytosis was also determined after washing the cultured medium away (**Figure 1C**). As compared to the efferocytosis capacity determined after 24 h in culture, the efferocytosis capacity of both types of macrophages decreased, if the culture medium was washed away (removal of the macrophage-produced bridging molecules), and phagocytosis was determined in the absence of fetal bovine serum (FBS) (removal of the serum-derived bridging molecules). What is more, the difference between the two types of macrophages completely disappeared. These data indicate that the enhanced efferocytic capacity of Nur77 KO macrophages is related either to enhanced production of a bridging molecule or to increased expression of a bridging molecule-dependent efferocytosis receptor. To determine, whether enhanced production of a bridging molecule is responsible for the observed effect, we compared the mRNA expression of each bridging molecule in the wild-type and Nur77 KO macrophages, and found that only that of MFG-E8 was increased, whereas no change in the mRNA expression of protein S, Gas6, C1qb or thrombospondin (THBS)-1 was found (**Figure 1D**). Next, we checked whether gene expression of the target efferocytosis receptors of the various bridging molecules is altered. However, we have not found a change in the expression of Axl, MerTK, or CD91, targets of Protein S, Gas6 or C1qb (**Figure 1E**). Since previous studies have shown that MFG-E8 binds to various integrin receptors *via* its RGD domain (8, 9), we also checked the mRNA expression of the various integrins and their coreceptors. While the mRNA expression of integrin β1 and β3, and that of Tim4 was not altered by the loss of Nur77 (**Figure 1E**), the basal gene expression of both CD36 and TG2 was significantly higher in the Nur77 KO macrophages, and we found a moderate increase in the expression of integrin β5 as well. Altogether, these data indicate that an enhanced integrin β3 and β5 signaling might be responsible for the enhanced efferocytosis capacity of Nur77 KO macrophages.

### Increased expression of PPARγ drives the enhanced phagocytic capacity of Nur77 KO BMDMs

**Figure 2:**
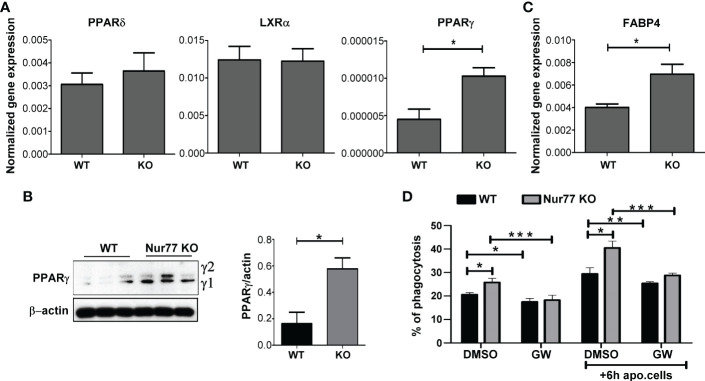
Loss of Nur77 leads to enhanced efferocytosis by BMDMs *via* upregulation of the PPARγ transcription factor. **(A)** mRNA expression levels of PPARδ, LXRα and PPARy in WT and Nur77 KO BMDMs following 24h in culture determined by qRT-PCR. β-actin was used as a reference gene. **(B)** Protein levels of PPARy in WT and Nur77 KO BMDMs under the same conditions determined by Western blot analysis. β-actin was used as a loading control. **(C)** mRNA expression levels of FABP4, a direct target gene of PPARγ, in WT and Nur77 KO BMDMs following 24h in culture detected by by qRT-PCR. β-actin was used as a reference gene. **(D)** Phagocytic capacity of WT and Nur77 KO BMDMs with or without pre-culture with non-labeled apoptotic thymocytes for 6 h, in the absence (DMSO control) or presence of PPARy antagonist GW9662 (5μM) for 24 h determined by flow cytometry. All results are expressed as mean ± SEM (n = 3). Asterisks indicate statistical significance (*P < 0.05, **P < 0.01, ***p < 0.001).

How can the loss of Nur77 affect the expression of genes related to the integrin β3 and β5 signaling pathways? Previous studies have shown that engulfing macrophages are capable of increasing their efferocytosis capacity *via* activation of their lipid-sensing nuclear receptors (liver X receptor (LXR)α/β, PPARγ and δ). These transcription factors, in turn, induce the expression of various phagocytic receptors or bridging molecules (26–28). Since the difference in the efferocytosis capacity between WT and Nur77 KO macrophages was more pronounced, if macrophages have already engulfed apoptotic cells (**Figure 1A**) when the content of the engulfed apoptotic material triggers activation of these nuclear receptors, we checked their mRNA expression. While the mRNA expression of PPARδ and that of LXRα did not change (**Figure 2A**), the mRNA expression of PPARγ significantly increased in Nur77 KO macrophages. Enhanced PPARγ levels were detected in the Nur77 KO macrophages at the protein level as well (**Figure 2B**). In addition, the mRNA level of its known target gene, fatty acid binding protein (FABP) 4 (29), was also significantly elevated in the Nur77 KO cells (**Figure 2C**). Accordingly, inhibition of PPARγ transcriptional activity by GW9662 for 24 h decreased the efferocytic capacity of Nur77 KO macrophages (**Figure 2D**). What is more, in the presence of the inhibitor the difference in the efferocytosis capacity of the two types of macrophages disappeared. Altogether these data indicate that the difference in the efferocytic capacity of the Nur77 KO macrophages is related to an enhanced PPARγ transcriptional activity.

### PPARγ induces the synthesis of retinoic acid to upregulate integrin signaling

**Figure 3:**
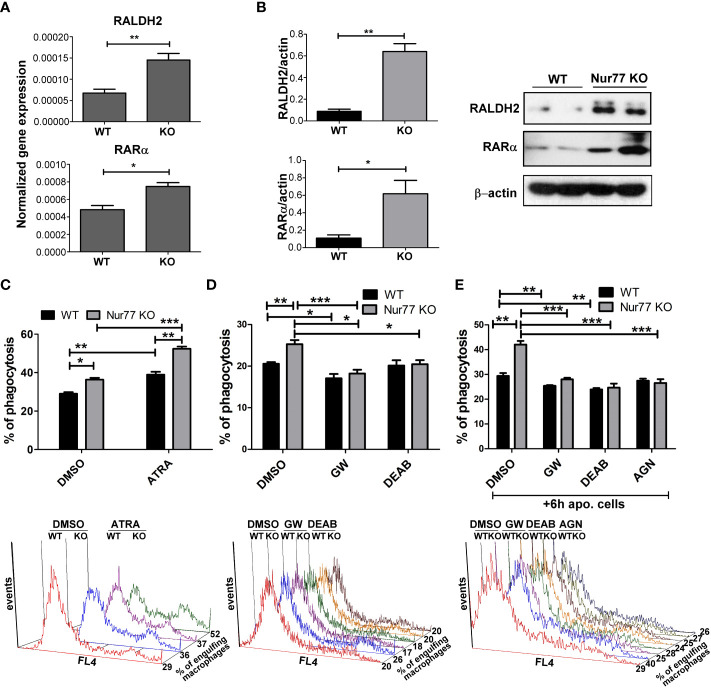
Elevated PPARγ expression leads to enhanced efferocytosis by Nur77 KO BMDMs *via* upregulating retinoic acid synthesis. **(A)** mRNA expression levels of RALDH2 and RARα of WT and Nur77 KO BMDMs following 24 h in culture determined by qRT-PCR. β-actin was used as a reference gene. **(B)** RALDH2 and RARα protein expression of WT and Nur77 KO BMDMs under the same conditions detected by Western blot analysis. β- actin was used as a loading control. **(C)** WT or Nur77 KO BMDMs were exposed to 300 nM ATRA or DMSO (vehicle) for 24 h, then the percentage of engulfing macrophages was determined by flow cytometry. A representative histogram of each experimental setting is also shown. **(D)** Wild-type and Nur77 KO BMDMs were exposed to GW9662 (5μM), a PPARγ antagonist, to DEAB (15 μM), an RALDH inhibitor, or to DMSO (vehicle) for 24h prior to determining their phagocytic capacity. The percentage of engulfing macrophages was detected by flow cytometry. A representative histogram of each experimental setting is also shown. **(E)** Wild-type or Nur77KO BMDMs were first exposed to non-fluorescent apoptotic thymocytes for 6 h together with GW9662 (5μM), with DEAB (15μM), or with the pan-RAR antagonist AGN193109 (1μM), then apoptotic cells were washed away and inhibitors was added again to BMDMs for additional 18 h prior to determining the percentage of macrophages engulfing apoptotic cells by flow cytometry. A representative histogram of each experimental setting is also shown. All results are expressed as mean ± SEM (n = 3). Asterisks indicate statistical significance (*P < 0.05, **P < 0.01, *** p < 0.001).

**Figure 4:**
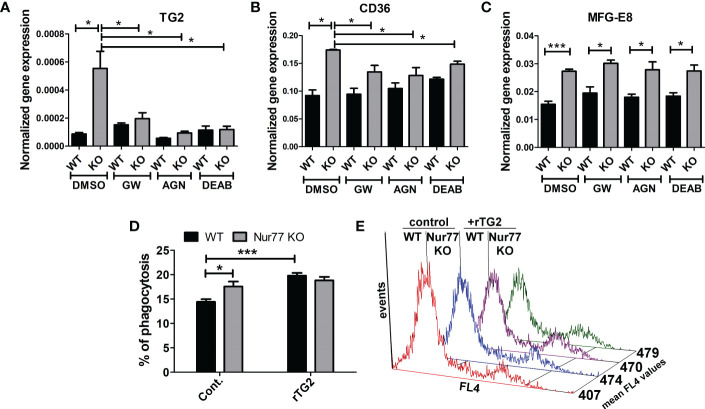
Increased efferocytosis capacity of Nur77 KO macrophages is strongly related to the enhanced TG2 expression. **(A-C)** mRNA expression levels of TG2, CD36 and MFG-E8 in wild-type and Nur77 KO BMDMs determined by qRT-PCR following a 24 h exposure to GW9662 (5 μM), DEAB (15 μM), AGN193109 (1 μM), or DMSO (vehicle). β-actin was used as a reference gene. **(D)** *In vitro* uptake of fluorescently-labeled apoptotic thymocytes by WT and Nur77 KO BMDMs after a 1 h exposure to recombinant mouse TG2 (rTG2) (10 μg/ml).The percentage of engulfing macrophages was determined by flow cytometry. **(E)** Mean fluorescence values of the engulfing macrophages in the above experiments indicating enhanced individual apoptotic cell uptake by the rTG2-exposed cells. Results are expressed as mean ± SEM (n = 3). Asterisks indicate statistical significance (*P < 0.05, **P < 0.01, ***p< 0.001).

Previous studies from our laboratory have demonstrated that engulfing macrophages produce retinoids that upregulate the expression of various phagocytic receptors (30, 31). This enhancement in the phagocytosis gene expressions was partially mediated *via* an LXR-dependent induction of retinaldehyde dehydrogenases (RALDHs), enzymes that are specifically involved in the synthesis of retinoic acids, and that of retinoic acid receptor (RAR)α, a nuclear receptor for which retinoic acids serve as ligands (31, 32). Since we have shown previously that rosiglitazone, a PPARγ agonist, was also capable of inducing RALDHs (30), we checked the mRNA and protein expression of RALDH2, the dominant RALDH in macrophages, and that of RARα. As seen **Figures 3A, B**, the expression of both RALDH2 and RARα was increased in Nur77 KO macrophages as compared to their wild-type counterparts at both mRNA and protein levels. Accordingly, Nur77 KO macrophages responded to all-trans retinoic acid (ATRA) treatment with a more significant increase in the efferocytosis capacity (**Figure 3C**). To determine whether PPARγ-regulated retinoid synthesis mediates the effect of the loss of Nur77 on the efferocytosis of macrophages, both WT and Nur77 KO macrophages were pre-treated for 24 h with the PPARγ antagonist GW9662, the RALDH inhibitor N,N-diethylaminobenzaldehyde (DEAB) or the pan-RAR antagonist AGN109, and their efferocytosis capacity was measured at the end of the treatments. These treatments decreased the efferocytosis capacity of both WT and Nur77 KO macrophages, and the efferocytosis capacity of Nur77 KO cells decreased to that of the WT ones (**Figures 3D, E**). Next we checked, how the above treatments affect the mRNA expression of those three phagocytosis genes, which we found to be significantly altered by the loss of Nur77 (**Figures 4A–C**). The most dramatic change was seen in the gene expression of TG2, in accordance with the observation that its expression was enhanced most by the loss of Nur77. While these treatments hardly affected the basal levels of TG2 in the WT macrophages, each treatment reduced the TG2 mRNA expression of Nur77 KO cells to the wild-type level. CD36 mRNA expressions, on the other hand, were only partially dependent on the PPARγ/retinoid signaling pathway, despite of the fact that CD36 expression was shown to be affected by both PPARγ and retinoids (33). Finally, the elevated MFG-E8 expression was independent of PPAR_γ_ indicating that Nur77 must suppress the expression MFG-E8 in WT cells *via* a different mechanism. Altogether, our data suggest that the enhanced PPARγ-dependent phagocytic capacity is largely dependent on the enhanced TG2 expression. TG2 was shown to facilitate integrin signaling and to promote efferocytosis added as a recombinant protein (11, 34). To determine whether addition of recombinant TG2 to WT macrophages could enhance their phagocytosis efficiency to the level of Nur77 KO cells, efferocytosis capacity of both cell types was determined after 24h in culture in the presence and absence of mouse recombinant TG2. As seen in **Figures 4D, E**, the addition of recombinant TG2 enhanced the phagocytic capacity of WT macrophages, but not that of the Nur77 KO cells. In addition, their phagocytic capacity was similar in the presence of recombinant TG2. These data provide further evidence for the TG2-dependence of the observed enhanced efferocytosis efficiency in Nur77 KO macrophages.

### Nur77 and PPARγ are expressed by distinct groups of M2 macrophages which drive skeletal muscle tissue repair following cardiotoxin injury

**Figure 5:**
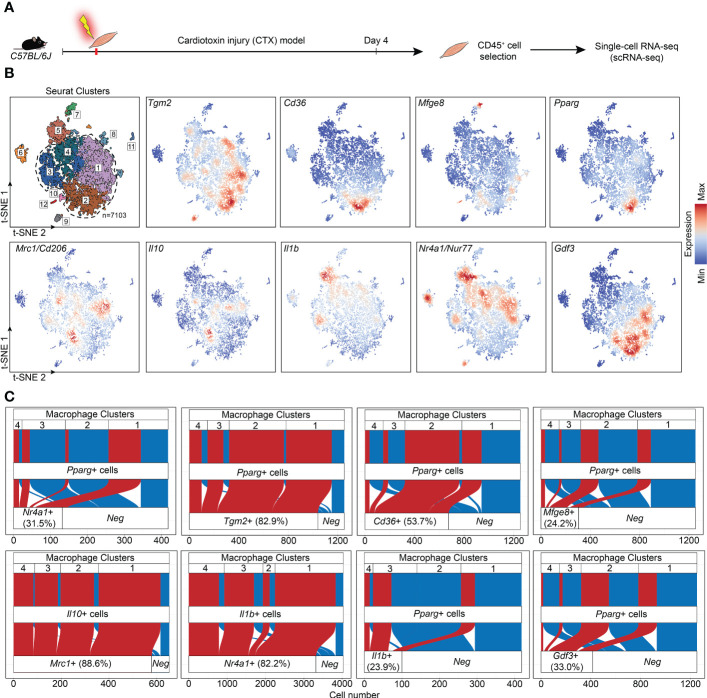
Immune single-cell transcriptomic map of regenerating murine skeletal muscle. **(A)** Workflow used for the isolation and analysis of previously published (39) single-cell RNAseq datasets from CD45^+^ cells isolated at day 4 following cardiotoxin-induced injury (see also “Methods”). **(B)** Upper left panel: unsupervised clustering and t-SNE representation of CD45+ myeloid cells isolated from Day 4 post CTX injury (colored by 12 clusters defined using cluster resolution 0.4; dotted line indicates the four macrophage clusters 1-4; 1: Resolution-related, 2: Growth Factor-Expressing, 3: Pro-inflammatory, 4: Antigen-presenting macrophages). The rest of the panels indicate *Tgm2, Cd36, Mfge8, Pparg, Mrc1, Il10, Il1b, Nr4a1*, and *Gdf3* mRNA expression in the single-cell dataset. **(C)** Alluvial plots that simultaneously define the number of cells with single and double positive expression for indicated genes and for each macrophage cluster. Red indicates cells with double positive/detectable gene expression in the third layer).

If Nur77 inhibits the expression of PPAR_γ_, how is it possible that apoptotic cell uptake was shown to activate both transcription factors in BMDMs (27, 35)? Increasing evidence indicate that macrophages do not form one single group of cells, and even within their two main groups there is a big heterogeneity. The first group, the tissue resident macrophages are mainly derived from the yolk sac during embryogenesis (1, 36). They act as sentinels in the tissues, and play an essential role in tissue homeostasis by removing apoptotic cells generated during the normal tissue turnover, and by producing growth factors and other mediators that provide trophic support to the tissues in which they reside. The second group, the BMDMs are recruited to the tissues in response to tissue injury induced by infection, autoimmune disorders, or by various injuries, and are crucial drivers and regulators of inflammatory and tissue regenerative responses (4, 37). One model, in which the differentiation, phenotype change (polarization) and function of BMDMs can be studied *in vivo*, is the cardiotoxin-induced injury model of the tibialis anterior skeletal muscle (7). Cardiotoxin injection triggers apoptotic and necrotic skeletal muscle cell death (38). Within this model, one day after the cardiotoxin injury monocytes arrive at the injury site and differentiate into M1 macrophages, which clear dead cells and, additionally, trigger muscle stem cell proliferation *via* the pro-inflammatory cytokines formed by them. As a result of the interaction with apoptotic cells during efferocytosis, these M1 macrophages start their conversion into M2-like reparative macrophages from day 3, and they drive angiogenesis, myotube formation from myoblasts, and the resolution of inflammation. Recent work by Patsalos et al. revealed the generation of three functionally distinct (growth factor producing, resolution-related and antigen presenting) populations of these M2-like reparative macrophages by analyzing their mRNA expressions using single-cell RNA sequencing at day 4 following cardiotoxin-induced injury (39). Using these data, we demonstrate in **Figure 5** that on a single cell level Nur77 and PPARγ are expressed by different groups of M2-like reparative macrophages, and even when they are co-expressed, the PPARγ levels are low. PPARγ-dominant expression characterizes the growth factor producing macrophage population, while Nur77 is mainly expressed by the resolution-related and antigen presenting cells. What is more, nearly all the PPARγ-expressing cells express TG2 or CD36 in the growth factor producing population (cluster 2), where the majority of high PPARγ-expressing macrophages were found, underlying our finding that PPARγ regulates their expression. However, only 24% of the PPARγ expressing macrophages express MFG-E8 (**Figure 5**), and only 11.2% of the MFG-E8-expressing cells express PPARγ (data not shown) in accordance with our *in vitro* finding that the increase in the MFG-E8 mRNA expression in Nur77 KO cells is largely PPARγ-independent.

### Loss of Nur77 leads to enhanced PPARγ and TG2 expressions, and to an accelerated CD206^+^ macrophage formation in day 4 skeletal muscle macrophages following cardiotoxin-induced injury

**Figure 6:**
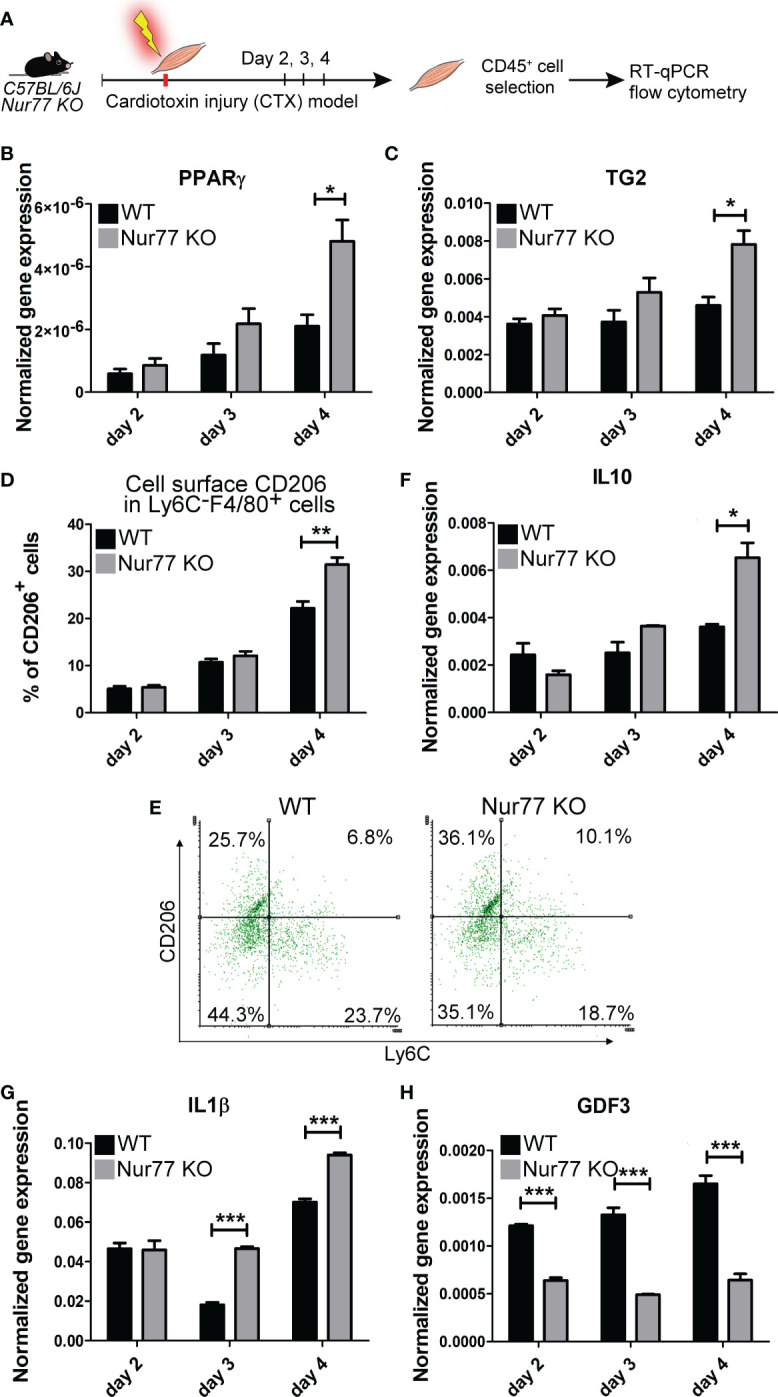
Contradicting M2 polarization of Nur77 KO skeletal muscle macrophages in the cardiotoxin-induced model of skeletal muscle injury. **(A)** Workflow used for the isolation and analysis of CD45^+^ cells isolated at the indicated days following cardiotoxin-induced injury in the tibialis anterior muscle. **(B, C, F-H)** Time-dependent alterations in the mRNA expressions of PPARγ, TG2, IL-10, IL-1β and GDF3 of CD45^+^ cells isolated from the tibialis anterior muscles determined by qRT-PCR at day 2, 3, and 4 post-injury (n= 4). β-actin was used as a reference gene. **(D)** Percent of CD206^+^cells within the Ly6C^-^ F4/80^+^ macrophages and **(E)** representative scatter plots of CD206^+^ and Ly6C^+^ cells within the muscle-derived F4/80^+^population determined at the indicated days following cardiotoxin-induced injury in the tibialis anterior muscles of wild-type and Nur77 KO mice (n= 3). Results are expressed as mean ± SEM. Asterisks indicate statistical significance (*P < 0.05, **P < 0.01, ***p< 0.001).

Our previous studies have demonstrated by studying macrophage polarization in the cardiotoxin-induced injury model of skeletal muscle that loss of TG2 not only affects the efficiency of the phagocytosis of dead cells, but also influences the expression levels of PPARγ, and the M1/M2 conversion of macrophages by significantly delaying the appearance of CD206^+^ M2-like reparative macrophage subset (40). Using the same model, we tested how the loss of Nur77 affects the expression of PPARγ, and TG2, and the appearance of CD206^+^ skeletal muscle macrophages following cardiotoxin-induced skeletal muscle injury. Simultaneously we also detected the disappearance of Ly6C, a marker, the disappearance of which allows us to follow the rate of M1/M2 macrophage conversion. As shown in **Figures 6A, B**, in Nur77 KO skeletal muscle-derived CD45^+^ cells the expression of both PPARγ and TG2 was significantly higher. Accordingly, the number of CD206^+^ macrophages was also significantly enhanced (**Figure 6C**). However, in line with a previous report (41), we did not find a difference in the rate of M1/M2 conversion of Nur77 KO skeletal muscle macrophages, if we followed it by the disappearance of the cell surface Ly6C molecule (**Figure 6D**). Interestingly, in TG2 null M2 macrophages the PPARγ expressions were found to be lower (40) indicating with the present data the existence of a positive autoregulatory loop that controls the expression of PPARγ or the number of PPARγ-expressing cells. PPARγ upregulates TG2, while the produced TG2 protein promotes either the stable expression of PPARγ mRNA or the better survival of the PPARγ-expressing macrophages. Altogether, our data indicate that the Ly6C^-^ PPARγ+ TG2^high^ macrophages are the origin of at least one group of the Ly6C^-^ CD206^+^ macrophage population. Since TG2 acts as a coreceptor for integrins, our observations confirm that of others, who have demonstrated the involvement of integrin signaling in the PPARγ-driven M2 conversion of macrophages (42, 43).

### Loss of Nur77 results in conflicting production of pro- and anti-inflammatory cytokines by skeletal muscle macrophages following cardiotoxin-induced injury

M1 and M2 macrophages are characterized by the production of a different set of cytokines. While M1 macrophages produce pro-inflammatory cytokines, such as TNF-α, IL-1α, IL-1β, IL-6, IL-12, CXCL9, and CXCL10, M2 macrophages produce IL-10, TGF-β, CCL1, CCL17, CCL18, CCL22, and CCL24 (44). During M1/M2 conversion the production of pro-inflammatory cytokines gradually decreases, while that of anti-inflammatory cytokines or growth factors increases. Since Nur77 was shown to suppress the expression of IL-1β (45), while PPARγ to induce IL-10 production (46), both contributing this way to the M2-like polarization, we decided to follow the mRNA expression of these cytokines in the skeletal muscle CD45^+^ cells following cardiotoxin-induced injury. As seen in **Figure 6E**, loss of Nur77 resulted in enhanced IL-10 production, a cytokine that plays a dominant role in promoting M2 conversion of the macrophages (47). Interestingly, we found that the producers of IL-10 are dominantly the CD206^+^ macrophages (**Figure 5**) suggesting that the observed enhanced IL-10 production is the consequence of an enhanced conversion of Nur77 KO M1 macrophages into the CD206^+^ direction by day 4. Altogether, these data demonstrate that PPARγ promotes IL-10 production by facilitating the formation of the IL-10-secreting CD206^+^ macrophages. Surprisingly, while the IL-10 production increased indicating an enhanced M2 polarization of Nur77 KO macrophages in the cardiotoxin model of skeletal muscle injury, the IL-1β mRNA levels were also enhanced (**Figure 6F**). This later indicates a prolonged inflammatory M1 state. However, as shown in **Figure 5**, IL-1β is produced by separate populations of macrophages, which express dominantly Nur77. This observation supports the view that Nur77 acts as a suppressor of IL-1β production.

### Loss of Nur77 results in decreased production of the growth differentiation factor 3

The way M2 macrophages are able to promote myoblast differentiation, myoblast fusion and myotube growth is that they produce various growth factors (39, 48, 49). Among them GDF3 was shown to be produced in a PPARγ-dependent manner (48). That is why we tested the expression of GDF3 in Nur77 KO skeletal muscle macrophages following cardiotoxin injury (**Figure 6G**). To our surprise, despite the higher expression of PPARγ, the expression of GDF3 was lower in Nur77 KO skeletal muscle macrophages (**Figure 6G**). Since the GDF3- and the PPARγ-expressing macrophage populations only partially overlap (**Figure 5**) (with 33% PPARγ-expressing cells expressing GDF3, and only 22.2% GDF3-expressing macrophages expressing PPARγ), our data indicate that Nur77 might contribute to the GDF3 expression in those Nur77^+^ macrophages, which do not express PPARγ. Accordingly, Nur77 KO BMDMs also showed about a 55% decrease in the GDF3 mRNA expression (data not shown), as reported by others as well (23).

## Discussion

**Figure 7:**
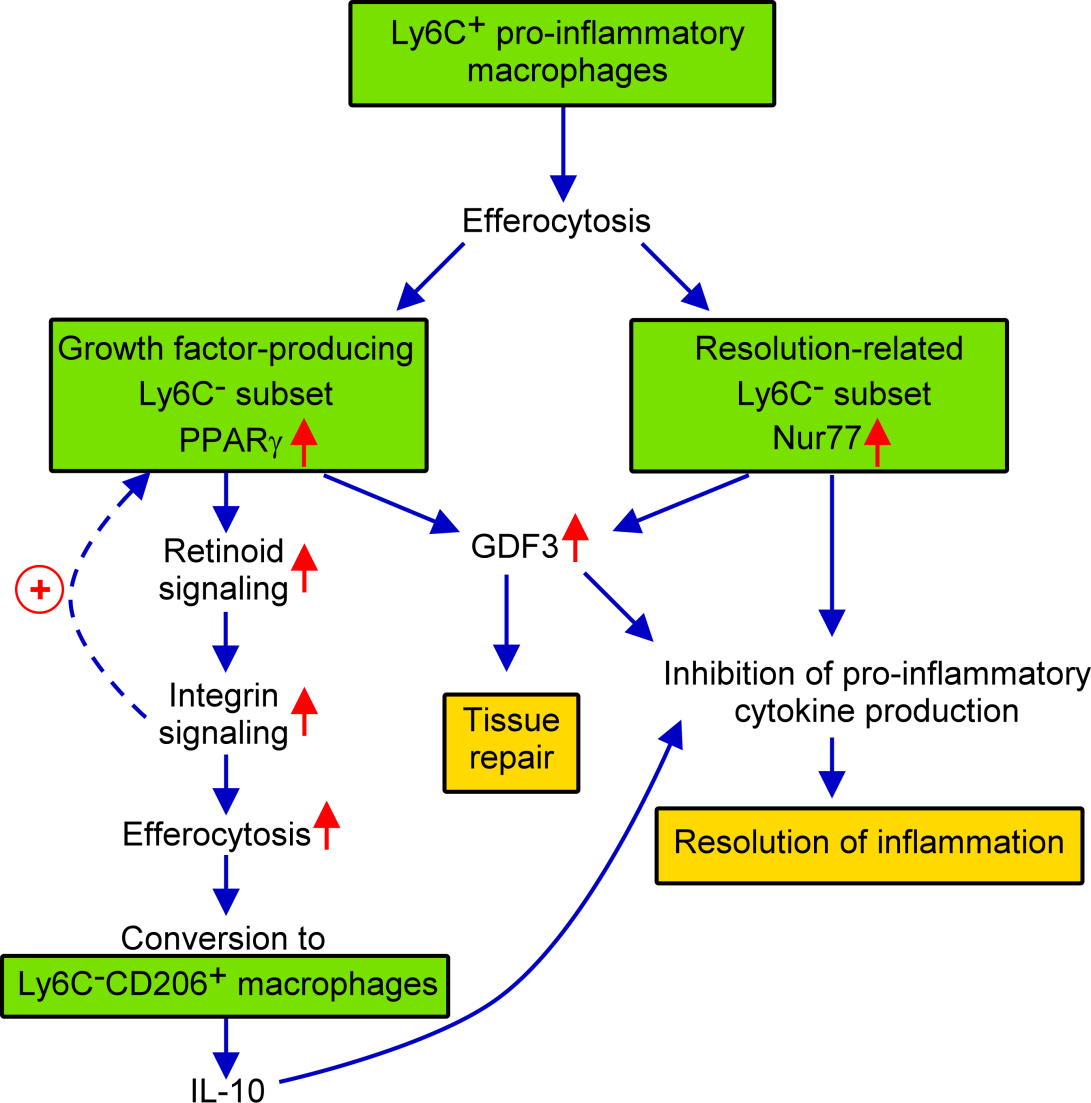
Schematic view of the events during conversion of M1 Ly6C^+^ pro-inflammatory macrophages to M2-like reparative macrophages following cardiotoxin induced skeletal muscle injury.

In the present paper the effect of the loss of Nur77 transcription factor was investigated on the *in vitro* efferocytosis function, and on the *in vivo* polarization of BMDMs in the cardiotoxin-induced skeletal muscle injury model. Using this model, we have shown previously that efferocytosis and polarization of macrophages are strongly associated phenomena (40, 50). Previous studies have indicated that in macrophages the expression and the transcriptional activity of both PPARγ and Nur77 are increased following apoptotic cell uptake (27, 35). Here we found that Nur77 KO BMDMs express increased amount of PPARγ leading to enhanced efferocytosis capacity. Increasing evidence indicates that Nur77 can repress the expression of PPARγ (51), while PPARγ that of the Nur77 (52). These observations might explain why Nur77 KO BMDMs express more PPARγ, and why these two transcription factors are expressed in distinct M2-like reparative skeletal muscle macrophage subsets following cardiotoxin injury. While PPARγ was known to promote phagocytosis of apoptotic cells (27, 53) and to facilitate M1/M2 conversion of macrophages (42, 43, 54), here we have demonstrated for the first time that its efferocytosis-related effects are mediated partly by upregulating retinoid signaling. Nur77 is known to act as a negative regulator of the inflammatory response (19, 21, 45). This effect of Nur77 is mediated partly by controlling the mitochondrial metabolism (45), but also by interfering with the NF-κB-mediated pro-inflammatory signaling (19). Our data confirm these observations by demonstrating enhanced IL-1β production by the normally Nur77-expessing macrophage subset. In addition, our data also demonstrate that Nur77 contributes to M2 polarization not only by inhibiting pro-inflammatory cytokine production but also by promoting GDF3 expression, since GDF3 is not only a growth factor, but also acts as a suppressor of the pro-inflammatory macrophage phenotype (55). Thus, we should expect an enhanced M1 polarization in the absence of Nur77. However, the polarization detected in the absence of Nur77 is conflicting which is related to the upregulation of PPARγ which drives enhanced M2 polarization in a different reparative macrophage subset. These contradictory effects might explain why some investigators found that loss of Nur77 promotes M1 polarization (22), while others have not (56). Our data also questions, how well the disappearance of Ly6C reflects the true nature of macrophage M2 polarization, since it was not affected in the absence of Nur77. The novelty of our findings is that we demonstrate for the first time that PPAR_γ_and Nur77 function in different subsets of macrophages, they appear in simultaneously differentiating macrophages in the cardiotoxin-induced skeletal muscle injury model, and their mutual antagonism makes sure that in one subtype the PPARγ-, while in the other subtype the Nur77-mediated transcriptional events dominate. We also demonstrate for the first time that PPARγ-expressing cells are the precursors of at least one of the CD206^+^ macrophage population, that dominantly the CD206^+^ macrophages are responsible for the IL-10 production, and that the production of GDF3 is controlled by both Nur77 and PPARγ. What we did not investigate was whether the loss of Nur77 also alters the size of the different subsets within the M2-like reparative population. Thus, increased PPARγ expression could also mean an increase in the size of the PPARγ expressing macrophage subset. The increase in the size of CD206^+^ macrophage population produced in a PPARγ-dependent manner indicates such a possibility. Still our data provide novel information about the heterogeneity of the bone marrow-derived macrophages, and about their polarization in a model of regenerative inflammation. Our results are summarized in **Figure 7**.

## Materials and methods

### Reagents

All reagents were obtained from Sigma-Aldrich (Budapest, Hungary) except when indicated otherwise.

### Experimental animals

The experiments were carried out with 4-week-old C57BL/6 or 2- to 4-month-old Nur77^+/+^ mice and their Nur77 deficient (57) littermates. Mice were maintained in specific pathogen-free condition in the Central Animal Facility, and all animal experiments were approved by the Animal Care and Use Committee of University of Debrecen (DEMÁB).

### Generation of apoptotic cells

Thymi from 4-week-old C57BL/6 mice were collected, and thymocytes were separated and incubated for 20 h (10^7^ cells/ml) in RPMI 1640 media supplemented with 2 mM glutamine, 100 U/ml penicillin and 100 μg/ml streptomycin. This treatment results in approximately 80% annexin V positive cells (58). Apoptotic thymocytes were then stained by 0.5 μM CellTrackerTM Deep Red dye (Invitrogen, Carlsbad, CA, USA) for 30 min in the absence of serum to be used later as target cells to detect efferocytosis by BMDMs.

### BMDM cell culture and treatments

2- to 4-month-old Nur77^+/+^ and Nur77^-/-^ mice were sacrificed by isoflurane overdose. Bone marrow progenitors were isolated from the femurs and tibiae by lavage with sterile physiological saline. Progenitor cells were differentiated to BMDMs in DMEM medium supplemented with 10% heat inactivated FBS, 20% conditioned medium derived from L929 cells, as a source for macrophage colony stimulating factor, 2 mM glutamine, 100 U/ml penicillin and 100μg/ml streptomycin for 5 days at 37°C in 5% CO_2_. On the third day, non-adherent cells were washed away. Following the 5-day culturing period macrophages were plated on cell culture plates for various treatments. For long-term apoptotic cell phagocytosis experiments non-labeled apoptotic thymocytes in 5:1 (apoptotic cells: macrophage) ratio were added to BMDMs for 6 h, then apoptotic cells were washed away and efferocytosis was determined 18 h later. In some experiments BMDMs were treated for 24 h with 5 μM GW9662 (Tocris Bioscience, Bristol, UK), a selective PPARγ antagonist, 1 μM AGN193109 (Tocris Bioscience, Bristol, UK), a pan-RAR antagonist, 15 μM DEAB to block aldehyde dehydrogenase enzyme activities or for 1 h with 10 μg/ml mouse recombinant TG2 protein prior to determining the phagocytic capacity. The dose of these compounds was the same used for BMDMs in the literature. None of these compounds affected the viability of macrophages tested by the appearance of cell surface PS detected by Annexin V-FITC binding.

### Western blot analysis

BMDMs were homogenized in ice-cold lysis buffer (10% v/v glycerol, 1% v/v Triton X-100, 1 mM EGTA, 20 mM Tris, pH 7.9, 100 μM β-glycerophosphate, 137 mM NaCl, 5 mM EDTA, 1.04 mM AEBSF, 0.8 μM aprotinin, 40 μM bestatin, 14 μM E-64, 20 μM leupeptin, and 15 μM pepstatin A). The protein content of the samples was determined by Bio-Rad Protein Assay Dye (Bio-Rad, Budapest, Hungary), and was diluted to 2 mg/ml, then the samples were boiled with an equal volume of Laemmli buffer. Electrophoresis was performed in 12% SDS-polyacrylamide gel. Separated proteins were transferred to an Immobilion-P transfer membrane (Millipore, Budapest, Hungary) and were probed overnight at 4°C with anti-mouse PPARy (81B8) (cat. #: 2443 Cell Signaling Technology, Beverly, MA, USA), RARα (sc-515796, Santa Cruz Biotechnology, Dallas, USA), RALDH2 (sc-393204, Santa Cruz Biotechnology, Dallas, USA) monoclonal antibodies in 1:1000 dilution, or monoclonal anti-β-actin antibody (A5441) in 1:5000 dilutions. After three washes with TBS-T, the membrane was incubated for 1 h with anti-mouse IgG (whole molecule)-peroxidase antibody produced in sheep or with goat-anti-rabbit IgG(H+L) HRP conjugate secondary antibody in 1:10000 dilutions followed by enhanced chemiluminescence (Advansta Inc., San Jose, CA, USA).

### *In vitro* apoptotic cell phagocytosis

Stained apoptotic thymocytes were added to the BMDMs in 5:1 (apoptotic cells/macrophage) ratio for 45 min for flow cytometry analysis or for 120 min for confocal microscopy. After co-culture, non-engulfed cells were washed away extensively, and macrophages were detached by trypsinization. The percentage of engulfing cells was determined on a Becton Dickinson FACSCalibur flow cytometer (Becton Dickinson Company, Franklin Lakes, NJ, USA).

### Confocal microscopy

For confocal microscopy, differentiated BMDMs were plated in 8-well chamber slides (8×10^5/^well) (IBIDI GmbH, Gräfelfing, Germany).). Phagocytosis assays were carried out as described above. After coculture, apoptotic cells were washed away and macrophages were stained with Alexa Fluor 488 conjugated anti-F4/80 antibody (MF48020, Invitrogen, Carlsbad, USA) for confocal microscopy analysis. Macrophages were then washed and fixed in 1% paraformaldehyde. Fluorescence confocal images were taken by a Zeiss fluorescent microscope (Zeiss LSM 880 confocal microscope Göttingen, Germany). Images were analyzed with ZEN 2012 v.1.1.0.0 software (Carl Zeiss Microscopy GmbH, Göttingen, Germany).

### Production of mouse recombinant TG2 protein

To obtain full-length recombinant N-terminally HIS_6_-tagged mTg2 protein, the coding part of mouse transglutaminase 2 cDNA (BC016492, Dharmacon, GE, Boston, MA, USA) was amplified and cloned into pET-30 Ek/LIC vector (Merck, Darmstadt, Germany) based on the manufacturer instructions using HPLC-purified 5’-GAC GAC GAC AAG ATG AGA ATT CAG ACC ATG GCA GAG GAG CTG C-3’ forward and 5’-GAG GAG AAG CCC GGT TGA ATT CGG TTA GGC CGG GCC GAT GAT AAC-3’ reverse primers. The cloning was confirmed by restriction digestion and Sanger sequencing (Eurofins Genomics, Ebersberg, Germany). Recombinant mTG2 protein was expressed in Rosetta 2(DE3) bacterial cells (Novagen, Merck, Germany) and purified as described previously using the improved protocol (59).

### Isolation of muscle-derived CD45^+^ leukocytes from the tibialis anterior muscle following cardiotoxin-induced injury

WT or Nur77 KO mice were anesthetized with 2.5% isoflurane using a SomnoSuite device. After anesthesia, muscle damage was induced in the tibialis anterior (TA) muscle by injecting 50 µL of 12 µM cardiotoxin (Latoxan, Valence, France) in phosphate-buffered saline (PBS). Mice were sacrificed and muscles were collected on days 2, 3, and 4 post-injury and processed for cell and mRNA analysis. TA muscles were dissociated in RPMI containing 0.2% collagenase II (Thermo Fisher Scientific, Waltham, MA, USA) at 37°C for 1 h and filtered through a 100 µm and then a 40 µm filter. Muscle-derived CD45^+^ cell isolation was carried out as described earlier (50). CD45^+^ cells were separated using magnetic sorting (Miltenyi Biotec, Gladbach, Germany).

### Single-cell RNA sequencing and analysis of muscle-derived CD45^+^ cells

Single-cell gene expression barcode, feature, and count matrices from CD45+ cells isolated on day 4 following cardiotoxin-induced muscle injury were used from dataset GSE161467 (39). Downstream analysis was carried out with R version 4.2.2 (2022–10–31). Quality control, filtering, data clustering, marker gene analysis, and visualization were carried out using Seurat (v4.0.3) R package with some custom modifications to the standard pipeline (see below) (60).Genes expressed in less than 5 cells and cells with a number of detected genes within the lower quantile (q0.975) were removed from the gene expression matrix. We removed any single cell with > 5% UMIs mapped to mitochondrial genes as well as doublets and outliers with UMI counts in the upper quantile (q97.5). After log-normalizing the data, the expression of each gene was scaled, and PCA was performed on the top 1000 most variable genes. Unsupervised shared nearest neighbor (SNN) clustering was performed with a resolution of 0.35, and visualization was done using t-distributed stochastic neighbor embedding (t-SNE) from *SCpubr* (61). Feature plots were generated using the *Nebulosa* package (62). The alluvial plots were generated using the *ggalluvial* package (a *ggplot2* extension) (63).

### Quantification of intramuscular immune cells by flow cytometry

The muscle-derived CD45^+^ cells, separated from Nur77^+/+^ or Nur77^-/-^ mice, were stained with a combination of Alexa Fluor 488-conjugated anti-F4/80 (MF48020, Invitrogen, Carlsbad, USA), PerCP-Cy5.5-conjugated anti-Ly6C (128012, BioLegend, San Diego, USA) and PE-conjugated anti-CD206 (141705, BioLegend, San Diego, USA) antibodies at room temperature for 15 minutes. Fluorescent intensity was detected with a Becton Dickinson FACSCalibur. Cells were gated based on their forward- and side-scatter characteristics. Macrophages were gated as F4/80^+^ cells. Fluorescent intensity was detected with a Becton Dickinson FACSCalibur.

### Analysis of mRNA expression

Total RNA was isolated from BMDMs or muscle-derived CD45^+^ cells by TRIzol (Invitrogen, Carlsbad, CA, USA) reagent according to the manufacturer’s guidelines. Total RNA was reverse transcribed into cDNA using High Capacity cDNA Reverse Transcription Kit (Life Technologies, Budapest, Hungary)according to the manufacturer’s instructions. Quantitative RT-PCR was carried out in triplicate using pre-designed FAM-labeled MGB assays (Life Technologies, Budapest, Hungary) on a Roche LightCycler LC 480 real-time PCR instrument. Relative mRNA levels were calculated using the comparative C_T_ method and were normalized to β-actin mRNA. Catalog numbers of the TaqMan assays used were the following: Actb Mm02619580_g1, integrin β1 Mm01253230_m1, integrin β3 Mm00443980_m1, integrin β5 Mm00439825_m1, CD36 Mm00432403_m1, MFG-E8 Mm00437836_m1, Protein S Mm01343426_m1, CD91 Mm00464608_m1, MERTK Mm00437221_m1, AXL Mm00500549_m1, transglutaminase 2 Mm00434920_m1, Thrombospondin-1 Mm00436979_m1, Tim4 Mm00449032_g1, CD14 Mm00432403_m1, PPARδ Mm00803184_m1, PPARγ Mm00440940_m1, LXRα Mm00443451_m1, RARα Mm01296312_m1, FABP4 Mm00445878_m1, RALDH2 Mm00501306_m1, GDF3 Mm00433563_m1, IL1 β Mm00434228_m1, and IL10 Mm01288386_m1.

### Statistical analysis

All the data are representative of at least three independent experiments carried out with BMDMs originated from three different mice or with mice exposed to cardiotoxin-induced injury. Values are expressed as mean ± SEM. For differences between 2 groups two-tailed unpaired Student’s t-test, for comparisons n > 2 groups one-way ANOVA (with Tukey’s multiple comparisons test) was used. The equal variance of the samples was tested by F-test. All statistical analyses were performed using GraphPad Prism 6.01 and a P value <0.05 was considered as significant, and is indicated by asterisk (*).

## Data availability statement

The datasets presented in this study can be found in online repositories. The names of the repository/repositories and accession number(s) can be found below:GSE161467 (GEO).

## Ethics statement

The animal study was reviewed and approved by Review Board of Animal Care and Use Committee of the University of Debrecen (DEMÁB) with permission numbers 7/2016/DEMÁB and 7/2021/DEMÁB.

## Author contributions

EG contributed to conception, design, data acquisition, analysis, and interpretation, EF, NT, TS-S, and ZSa, contributed to data acquisition, AP and LH contributed to data analysis, AP also critically revised the manuscript. RK provided material, ZSz contributed to conception, design and data interpretation, drafted the manuscript. All authors read and approved the final manuscript.
